# Formation of Nanochannels Using Polypropylene and Acetylcellulose for Stable Separators

**DOI:** 10.3390/membranes12080764

**Published:** 2022-08-04

**Authors:** Hye Ji Lee, Younghyun Cho, Sang Wook Kang

**Affiliations:** 1 Department of Chemistry, Sangmyung University, Seoul 03016, Korea; 2 Department of Energy Systems Engineering, Soonchunhyang University, Asan 31538, Korea; 3 Department of Chemistry and Energy Engineering, Sangmyung University, Seoul 03016, Korea

**Keywords:** separator, battery, thermal stability, cellulose, channel

## Abstract

In this study, a polymer separator with enhanced thermal stability is prepared to solve the problem of thermal durability of lithium-ion battery separators. This separator is manufactured by coating a solution of acetyl cellulose and glycerin on polypropylene. The added glycerin reacts with the acetyl cellulose chains, helping the chains become flexible, and promotes the formation of many pores in the acetyl cellulose. To improve the thermal stability of the separator, a mixed solution of acetyl cellulose and glycerin was coated twice on the PP membrane film. Water pressure is applied using a water treatment equipment to partially connect the pores of a small size in each layer and for the interaction between the PP and acetyl cellulose. SEM is used to observe the shape, size, and quantity of pores. TGA and FT-IR are used to observe the interactions. Average water flux data of the separators is 1.42 LMH and the decomposition temperature increases by about 60 °C compared to the neat acetyl cellulose. It is confirmed that there is an interaction with PP between the functional groups of acetyl cellulose.

## 1. Introduction

As the awareness of environmental problems around the world increases, the interest in eco-friendly and sustainable products has been increasing. Thus, these changes have affected the eco-friendly battery industry. There is increasing demand for lithium-ion batteries in a variety of industries, particularly in the field of electric vehicles and small mobile devices [1,2]. The fast-growing battery electric vehicle (BEV) industry over the past five years in Europe, China, and the United States [3,4,5], and the high utilization and development of electronic devices around the world are related to the growth of lithium-ion batteries [6,7]. Lithium-ion batteries have been a key topic for many researchers in recent years. In particular, many studies are being conducted to improve the energy density, which is the core of lithium-ion batteries technology [8,9,10,11]. However, the stability problem caused by the battery separator is still an unavoidable problem [12,13,14,15,16]. The battery separator is one of the main factors influencing the safety since it directly contributes to the thermal stability of the overall battery system. Although there is no direct chemical reaction within the separator, the structure and properties of the separator play an essential role in determining the battery performance [17,18,19]. The role of separators in lithium-ion batteries can be roughly summarized into four categories. First of all, the separator separates the positive and negative electrodes from the inside of the battery. Second, the separator has fine pores and it plays a role in allowing lithium ions to move between the anode and the cathode through the pores. Third, when the internal temperature of the battery increases above a certain level, it blocks the movement of lithium ions through the pores located on the surface of the separator to prevent an internal short circuit. Lastly, the separator has high mechanical strength for securing safety when subjected to strong force.

Since the separator is related to the safety of the battery, several conditions are required. First of all, for use as a separator, it must be electrochemically stable and show excellent insulation. This is because the contact between the anode and the cathode must be cut off. In addition, the separator must have many pores so that lithium ions can pass through between the separators, and the size should be uniform. If the size of the pores is irregular, it causes the movement of lithium ions to be difficult. In addition, when the temperature of the battery exceeds above a certain level, the separator must show shut down to block the movement of lithium ions to ensure safety. Lastly, in order to improve the energy density of battery, a larger amount of active material can be loaded when the battery is thin, and the mechanical strength of the separator must be excellent so that it is not easily collapsed.

Therefore, recently, studies to improve the various performances of these separators have been conducted. Peng et al. designed a heat/fire-resistant dual-function separator by coating ammonium polyphosphate on a ceramic-coated separator modified with phenol-formaldehyde resin and tested it for 30 s at a temperature of 300 °C or higher [20]. Ahn et al. significantly reduced costs by using a water-soluble binder to coat reactive Al_2_O_3_ particles on polyethylene (PE) separators and achieved the uniform coating to develop separators for good thermal safety and cycle performance at high temperatures [21]. Costa et al. manufactured poly (vinylidene fluoride-co-trifluoro ethylene) separator film by removing ZnO nanoparticles from polymer matrix complexes. After 70% removal of ZnO, the membrane manufactured showed improved speed and cycling performance [22]. Prasanna et al. manufactured the dip-coated polyethylene separator with NiO. Compared to PE in the neat state, it had a high ion conductivity of 2.12 mS cm^−1^, with approximately 6 times lower enthalpy values, lower weight loss, and lower heat shrinkage behavior even when exposed to high temperatures [23]. Zhang et al. succeeded in making a lithium-ion battery separator with excellent heat resistance and renewable cellulose-based nonwoven fabric through electrospinning technology and the dip coating process. By manufacturing a cellulose/PVDF-HFP composite separator having excellent electrolyte wettability, the excellent heat resistance and high ionic conductivity, a new method for application to lithium-ion batteries was proposed [24]. Yanilmaz et al. have successfully fabricated a SiO_2_/PAN nanofiber separator using sol-gel and electrospinning methods. The SiO_2_/PAN hybrid nanofiber membrane showed higher ionic conductivity, thermal stability, and lower interfacial resistance than the microporous PP membrane. In addition, by increasing the SiO_2_ contents, the electrolyte absorption capacity and ionic conductivity were promoted, leading to excellent C-rate performance and cycling of cells using these membranes [25].

Our research group has also tried various attempts to improve the performance of the separator. Acetyl cellulose (AC) polymer was selected as the main material for these polymer membranes [26,27,28,29]. Cellulose derivatives refer to products obtained by oxidation, substitution, and other chemical treatment of wood-based materials, and are used as raw materials in various chemical industries depending on the type of functional group to be substituted. Various derivatives can be obtained by substitution by esterification or etherification, and the derivatives include cellulose nitrate, acetyl cellulose, and methyl/ethyl cellulose. Among them, AC, a cellulose ester series, has been mass-produced since the 1930s and has been used to replace cellulose nitrate. As a sufficiently commercialized product, it has the advantage of being inexpensive and the main raw material is eco-friendly [30,31,32].

In general, the most common method for forming pores in a separator is a method using radiation and phase separation. Radiation is advantageous in forming straight-like channels. However, the process is very disadvantageous in terms of price. Thus, separators have been mainly prepared through phase separation. However, in the case of using the phase separation method, it is difficult to form uniform size pores and straight-like channels. Therefore, we conducted a study on pore formation through hydraulic pressure and additives such as wetting agents and metal salts. This method has the advantage that it can be reused in the process if the used water goes through a filter, and the amount and size of pores can be easily adjusted by the number of additives or water pressure [26,27,28,29]. Since metal salts are more expensive than wetting agents, glycerin was used in this study. In addition, in order to improve the thermal stability of the separator, an AC polymer solution containing glycerin was coated on a nanoporous polypropylene (PP) support. Through these materials, we proposed a method to interconnect the nanopores of AC/glycerin and PP with only solvent and water pressure without adhesive.

## 2. Materials and Methods 

### 2.1. Materials

For preparation of polymer solution, acetyl cellulose (AC, Mw 30,000) was purchased from Sigma-Aldrich. Acetone (99.8%) and glycerin (Gly, 99%) were purchased from Daejung chemicals and metals Co., Ltd., Seoul, Korea. Polypropylene membrane filter (PP, pore size avg.: 100 nm; diameter: 90 mm; thickness: 110 μm) as a support was purchased from GVS KOREA., Ltd., Namyangju-si, Gyeonggi-do, Korea (disk and sheet type filters model No. 1220824).

### 2.2. Methods

#### 2.2.1. Separator Preparation

**Figure 1:**
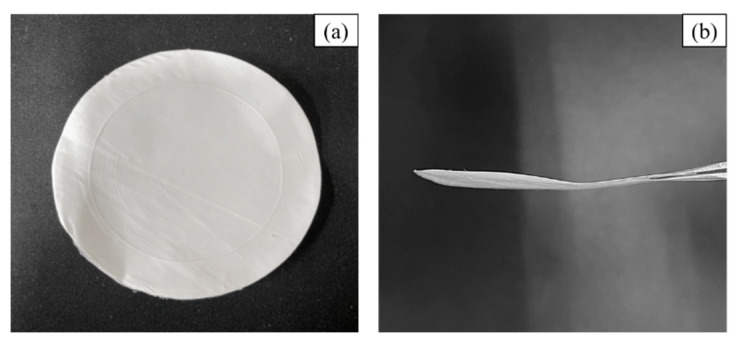
Appearance of the manufactured PP/AC/Gly separator. (**a**) Top view of the separator, (**b**) side view of the separator.

A 10 wt% AC/Gly solution was prepared by mixing 0.05 mol of glycerin and 1 g of acetyl cellulose. The 0.05 mol of glycerin was inserted to monomeric 1 mol of AC, and cosolvent (acetone:distilled water 8:2 wt%) was used as the polymer solvent. This AC/Gly solution was stirred for 15 h (25 °C and 50%). The AC/Gly solution was cast on the PP film using a blade with a thickness of 300 μm. Then, the film was dried in a thermo-hygrostat for 20 min. After that, the AC/Gly solution was coated once more on the PP film coated with AC/Gly solution to a thickness of 300 μm. Then, it was dried in a thermo-hygrostat for 20 min. Water pressure was applied to the completed PP/AC/Gly separators for 1.5 h at 8 bar, and then the pressure increased to 10 bar for 3.5 h. A total of 5 h of water pressure formed water channels in the polymers and induced the adhesion between the PP film and AC/Gly film (Figure 1). The flux data were measured as L m^−2^ h^−1^ (LMH).

#### 2.2.2. Characterization

The separators used during all the analysis procedures were vacuum dried for 3 days to completely remove the solvent before use. The water flux was measured using a hydraulic treatment machine (test cell system). The test cell equipment is specially manufactured, and the following paper can be referred to for a detailed description of the equipment [30]. A scanning electron microscope (SEM, JSM-5600LV, JEOL, Tokyo, Japan) was used to observe the surface state of the separator. Thermogravimetric analysis (TGA, Universal V4. 5A, TA instruments, Mettler Toledo, Columbus, OH, USA) and Fourier transform infrared spectroscopy (FT-IR, VERTEX 70V FT-IR spectrometers, Bruker, Billerica, MA, USA) were used to observe the interconnected bonding of films and thermal stability. 

## 3. Results and Discussion

### 3.1. Water Flux Data

In this experiment, the PP side was exposed to water first, and water flowed towards the AC/Gly side. After applied to a water pressure of 8 bar for 1.5 h, the weakened area of composites started to form the pores. To ensure reproducibility, the complete water channels were created by exposing to 10 bar for 3.5 h. If only 1.5 h of water pressure is applied at 8 bar, the flux data is not uniform. However, reproducible flux data could be obtained by applying water pressure for an additional 3.5 h at 10 bar. This phenomenon is expected to connect some pores at a pressure of 8 bar and form the complete water channels at a pressure of 10 bar. As shown in Table 1, in the case of neat PP, there are abundant pores and the membrane itself is thin. Thus, when it is fastened to water treatment equipment, water flows so rapidly that it cannot measure the flux. On the other hand, when neat AC (acetone:H_2_O = 8:2, no additives) was coated twice, no water came out at all. Under these circumstances, the PP/AC/Gly separator showed an average of 1.42 (±0.5) LMH, and reproducible results were obtained. It was found that the water channels were successfully created by connecting the pores of the existing PP film with the pores of the AC/Gly film through the two layers. Since proper pore formation through which lithium ions can move is essential for the separator, reproducible pore formation through additives is expected to be meaningful in this experiment.

**Scheme 1:**
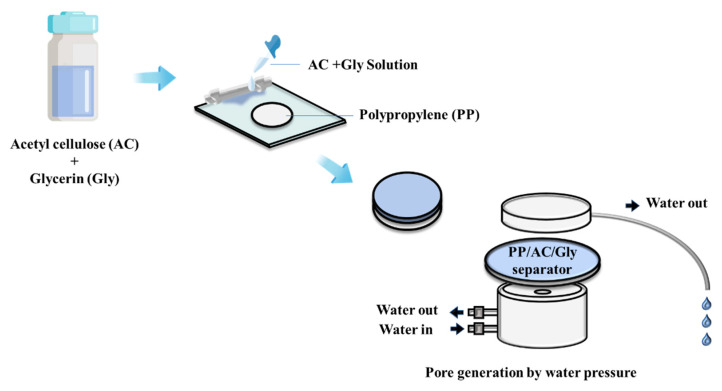
Preparation process of the PP/AC/Gly separator.

Scheme 1 described the manufacturing process of separator with water treatment equipment. To be specific about the water treatment equipment in Scheme 1, the separator was placed in the equipment and screwed up securely. Therefore, water with constant pressure from the bottom to the top cannot go out without going through the separator. If no channels are formed in the separator, the water flows back through the pipe below towards the inlet (in Scheme 1: ‘Water out’ at the bottom). However, if a water channel is formed in the separator, the water that comes out through the separator comes out through the external pipe (in Scheme 1: ‘Water out’ at the top).

### 3.2. SEM Images

**Figure 2:**
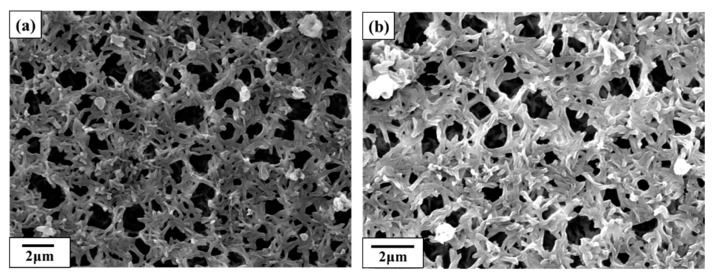
Images of (**a**) the neat PP film and (**b**) the PP side of the PP/AC/Gly at 10 bar.

**Figure 3:**
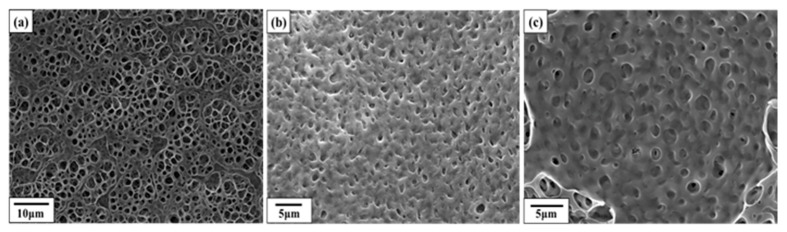
Images of (**a**,**b**): the upper surfaces of AC/Gly film before and after water pressure treatment, respectively, and (**c**) the AC side of the PP/AC/Gly separators at 10 bar.

For the PP films (average pore size = 100 nm) purchased from the manufacturer, large pores of 2 μm were also observed in SEM. In addition, the SEM image supported that the flux value of neat PP is so high that it cannot be measured. Using these films, PP/AC/Gly separators were prepared, and water pressure was applied up to 10 bar. When the PP side was observed (Figure 2b), there was no change in the shape of the chains. However, the side of acetyl cellulose was different. After adding glycerin to neat AC and dried (Figure 3a), the cosolvent (acetone and distilled water) was vaporized and there were pore-like substances. It was thought that pores were not generated inside since it is in the state before applying water pressure. When the water pressure was applied up to 10 bar (Figure 3a), the upper surface of polymers was slightly changed by water pressure (Figure 3b), and the channels were formed inside. When the AC/Gly solution prepared was coated on PP and observed toward AC (Figure 3c), some pores were connected to form new water channels.

In detail, the glycerin as an additive has three hydroxyl groups, and thus it could be well hydrated with water. It is dispersed among the dense chains of acetyl cellulose, making it flexible by increasing the distance between the polymer chains. AC/Gly solution was coated on PP film, and when water pressure was applied to this separator, the additive came out together with water and pores were formed in acetyl cellulose. In addition, due to the physical pressure caused by water pressure, the PP chains and AC chains were entangled and strongly bonded to each other, and at the same time, pores were connected to form water channels. With this method, it was possible to form nanochannels by connecting microscopic nano-sized pores without a separate adhesive.

### 3.3. TGA Data

**Figure 4:**
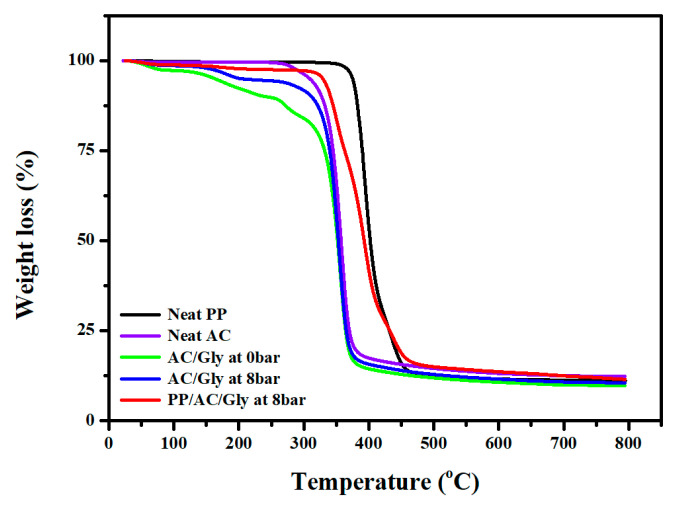
TGA data of neat PP, AC film, AC/Gly, and PP/AC/Gly separators.

TGA data in Figure 4 showed that the decomposition of neat AC and neat PP started at about 265 and 350 °C, respectively. In the case of a film prepared by mixing neat AC with glycerin (AC/Gly at 0 bar), the polymer chains became flexible due to the hydration effect of the OH functional groups of glycerin, and thus the rapid weight change from 140 °C was observed. However, the glycerin removed by the physical pressure of water of 8 bar was decomposed at a temperature higher than 140 °C (AC/Gly at 8 bar). On the other hand, in the case of PP/AC/Gly separators, the decomposition started at a higher temperature (325 °C) than neat AC. Water pressure could induce the surface adhesion between AC/Gly chains and PP chains, and it was presumed to cause the crosslinking effects between the two polymer films. These phenomena were consistent with previous studies [33]. Polyethylene, a lithium-ion battery separator material that is currently commercially available, shows the shut-down at 130 °C and completely molten at 150 °C. In the case of polypropylene, it is only about 15 °C higher. As this temperature increases, the internal short circuit of the battery can be prevented. Thus, by coating AC (T_g_: 160–180 °C, T_m_: 230–300 °C) on PP, AC can serve as a support even when PP starts to become molten. The TGA measurement data show indirectly that the thermal stability was not significantly reduced despite the plasticization of acetyl cellulose through additives, and thermal stability was maintained even when combined with PP.

### 3.4. FT-IR Data

**Figure 5:**
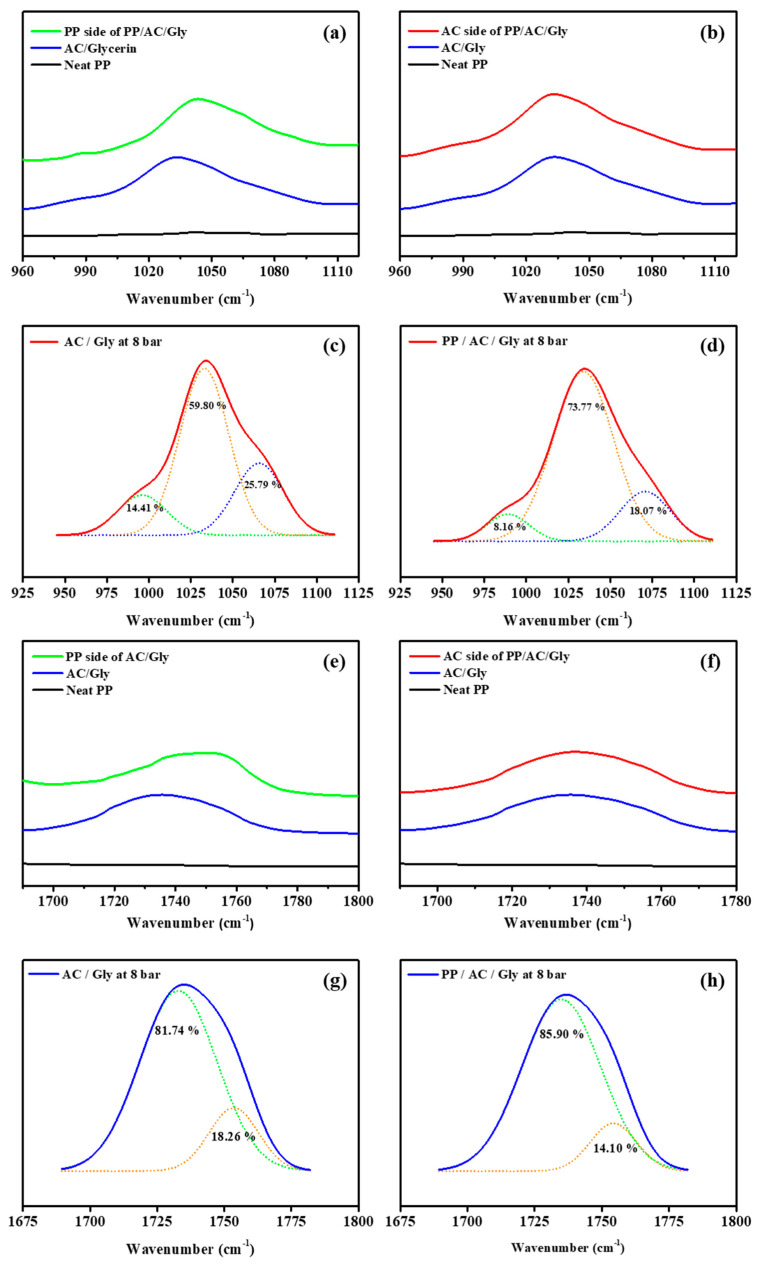
(**a**,**b**): FT-IR spectra for ether (960–1100 cm^−1^), (**c**,**d**) deconvoluted data of the ether functional group (960–1100 cm^−1^) of the PP/AC/Gly separators, (**e**,**f**) FT-IR spectra for the carbonyl functional group (1690–1780 cm^−1^) of the PP/AC/Gly separators, and ((**g**,**h**) and Table 3) deconvoluted data of the carbonyl functional group (1690–1780 cm^−1^) of the PP/AC/Gly separators.

As a result of FT-IR analysis for ether groups in the 960–1100 cm^−1^ on the PP side of the PP/AC/Gly separator, it was clearly confirmed that it moved to a higher wavenumber than the AC/Gly film (Figure 5a,b). It can be inferred that the AC/Gly film was coated on the PP and the new interactions in the functional groups affected the existing ether groups. When the deconvolution was performed on the AC side, the peaks tended to be symmetric at 1034 cm^−1^ in the PP/AC/Gly separator, compared to the AC/Gly film in which peaks were widely distributed at 8 bar. (Figure 5c,d and Table 2). These results could also be presumed to be due to the new interactions with PP in the ether functional groups.

In the case of the carbonyl functional groups, it clearly moved to a higher wavenumber from the PP side when the PP/AC/Gly separator was prepared as in the case of ether functional group analysis (Figure 5e,f). When deconvolution was performed on the AC side (Figure 5g,h), it was confirmed that it moved about 4.16% to the lower wavenumber than AC/Gly film at 8 bar. This demonstrated that PP formed new interactions with the carbonyl groups of AC, and that these interactions could generate the cross-linking as shown in TGA.

### 3.5. Electrochemical Performance

This study was carried out as a series of studies conducted by our research team. Referring to our previous research, the excellent electrochemical properties of acetyl cellulose were observed [34]. An LTO/AC separator (after hydraulic treatment)/Li metal composition battery with 1.3M LiPF_6_ of EC/DEC (50 *v*/50 *v*) with 10% FEC was constructed. As a result, the stable performance of discharging/charging plateaus at 1.54 and 1.58 V was measured. In addition, when various current rates from 1 to 15 C using this half-cell were measured, the cell’s current rate monotonically decreased for the average capacity from 160 to 50 mAh/g and recovered to the 1C rate [34]. These data can prove the electrochemical stability of the cellulose materials we are currently developing. Thus, similar results are expected to be observed at the PP/CA/Gly separator since PP is just utilized as support.

## 4. Conclusions

**Scheme 2:**
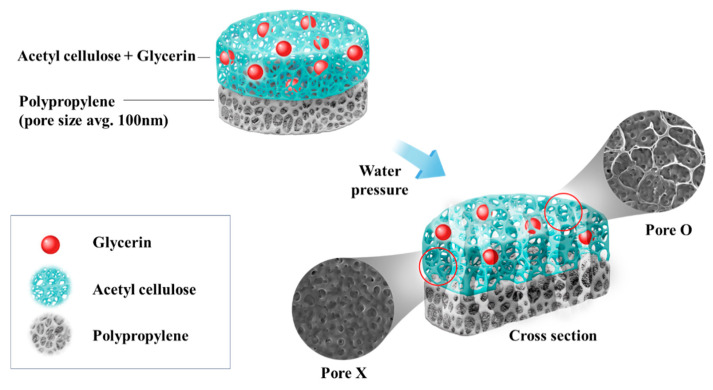
Formation of PP/AC/Gly separators.

In this study, the stable battery separator was prepared to solve the stability problem. The main purpose was to prepare a stable separator using an inexpensive material as well as nano-sized pores connected without a separate adhesive. As shown in Scheme 2, a polypropylene (PP) film, a widely commercially available material, was used as the support layer, and acetyl-cellulose containing glycerin was coated on PP. Since glycerin used as an additive has three -OH functional groups at one molecule, it was effectively hydrated on the AC chains, and it could generate the plasticizing effect to easily form pores. When water pressure was applied to the PP/AC/Gly separators, the carbonyl group and ether group of AC caused new physical interactions with the PP chains. Furthermore, the adhesion was generated and nano-sized pores were connected between the two polymer layers. In addition, when compared with the existing neat AC, the PP/AC/Gly separators were able to prove that the thermal stability was improved, confirmed by measuring the decomposition temperature increased by about 60 °C as well as the enhanced mechanical property by PP support.

## Figures and Tables

**Table 1:** Water flux data of separators.

	Neat AC at 10 Bar	Neat PP at 10 Bar	PP/AC/Gly at 10 Bar
LMH	0	Not measurable	1.42 (±0.5)

**Table 2:** Deconvoluted % of FT-IR data (ether functional group).

Peak (cm^−1^)	Area (%)	
	AC/Gly	PP/AC/Gly
989–996	14.41	8.16
1033–1034	59.80	73.77
1066–1071	25.79	18.07

**Table 3:** Deconvoluted % of FT-IR data (carbonyl functional group).

Peak (cm^−1^)	Area (%)	
	AC/Gly	PP/AC/Gly
1732	81.74	85.90
1753	18.26	14.10

## Data Availability

Not applicable.

## References

[1] Costa C.M., Barbosa J.C., Gonçalves R., Castro H., Campo F.J.D., Lanceros-Méndez S. (2021). Recycling and environmental issues of lithium-ion batteries: Advances, challenges and opportunities. Energy Storage Mater..

[2] Yang Y., Okonkwo E.G., Huang G., Xu S., Sun W., He Y. (2021). On the sustainability of lithium ion battery industry—A review and perspective. Energy Storage Mater..

[3] Wen W., Yang S., Zhou P., Gao S.Z. (2021). Impacts of COVID-19 on the electric vehicle industry: Evidence from China. Renew. Sustain. Energy Rev..

[4] Pan S., Fulton L.M., Roy A., Jung J., Choi Y., Gao H.O. (2021). Shared use of electric autonomous vehicles: Air quality and health impacts of future mobility in the United States. Renew. Sustain. Energy Rev..

[5] Haustein S., Jensen A.F., Cherchi E. (2021). Battery electric vehicle adoption in Denmark and Sweden: Recent changes, related factors and policy implications. Energy Policy.

[6] Olabi A.G., Adil M., Sayed E.T., Iqbal A., Rodriguez C., Abdelkareem M.A. (2021). Lithium-Ion Batteries. Anonymous Reference Module in Materials Science and Materials Engineering.

[7] Scrosati B. (2000). Recent advances in lithium ion battery materials. Electrochim. Acta.

[8] Jamil S., Yousaf A.B., Yoon S.H., Han D.S., Yang L., Kasak P., Wang X. (2021). Dual cationic modified high Ni-low co layered oxide cathode with a heteroepitaxial interface for high energy-density lithium-ion batteries. Chem. Eng. J..

[9] Casino S., Niehoff P., Börner M., Winter M. (2020). Protective coatings on silicon particles and their effect on energy density and specific energy in lithium ion battery cells: A model study. J. Energy Storage.

[10] Zoller F., Böhm D., Luxa J., Döblinger M., Sofer Z., Semenenko D., Bein T., Fattakhova-Rohlfing D. (2020). Freestanding LiFe0.2Mn0.8PO4/rGO nanocomposites as high energy density fast charging cathodes for lithium-ion batteries. Mater. Today Energy.

[11] Xia L., Miao H., Zhang C., Chen G.Z., Yuan J. (2021). Review—Recent advances in non-aqueous liquid electrolytes containing fluorinated compounds for high energy density lithium-ion batteries. Energy Storage Mater..

[12] Balakrishnan P.G., Ramesh R., Kumar T.P. (2006). Safety mechanisms in lithium-ion batteries. J. Power Sources.

[13] Bandhauer T.M., Garimella S., Fuller T.F. (2011). A critical review of thermal issues in lithium-ion batteries. J. Electrochem. Soc..

[14] Liu K., Liu Y., Lin D., Pei A., Cui Y. (2018). Materials for lithium-ion battery safety. Sci. Adv..

[15] Huang W., Feng X., Han X., Zhang W., Jiang F. (2021). Questions and Answers Relating to Lithium-Ion Battery Safety Issues. Cell Rep. Phys. Sci..

[16] Sun P., Bisschop R., Niu H., Huang X. (2020). A review of battery fires in electric vehicles. Fire Technol..

[17] Li A., Yuen A.C.Y., Wang W., Cordeiro D., Miguel I., Wang C., Chen T.B.Y., Zhang J., Chan Q.N., Yeoh G.H. (2021). A Review on Lithium-Ion Battery Separators towards Enhanced Safety Performances and Modelling Approaches. Molecules.

[18] Lee H., Yanilmaz M., Toprakci O., Fu K., Zhang X. (2014). A review of recent developments in membrane separators for rechargeable lithium-ion batteries. Energy Environ. Sci..

[19] Costa C.M., Lanceros-Mendez S. (2021). Recent advances on battery separators based on poly(vinylidene fluoride) and its copolymers for lithium-ion battery applications. Curr. Opin. Electrochem..

[20] Peng L., Kong X., Li H., Wang X., Shi C., Hu T., Liu Y., Zhang P., Zhao J. (2021). A Rational Design for a High-Safety Lithium-Ion Battery Assembled with a Heatproof–Fireproof Bifunctional Separator. Adv. Funct. Mater..

[21] Ahn J.H., Kim H., Lee Y., Esken D., Dehe D., Song H.A., Kim D. (2021). Nanostructured reactive alumina particles coated with water-soluble binder on the polyethylene separator for highly safe lithium-ion batteries. J. Power Sources.

[22] Costa C.M., Kundu M., Dias J.C., Nunes-Pereira J., Botelho G., Silva M.M., Lanceros-Méndez S. (2019). Mesoporous poly(vinylidene fluoride-co-trifluoroethylene) membranes for lithium-ion battery separators. Electrochim. Acta.

[23] Prasanna K., Subburaj T., Lee W.J., Lee C.W. (2014). Polyethylene separator: Stretched and coated with porous nickel oxide nanoparticles for enhancement of its efficiency in Li-ion batteries. Electrochim. Acta.

[24] Zhang J., Liu Z., Kong Q., Zhang C., Pang S., Yue L., Wang X., Yao J., Cui G. (2013). Renewable and superior thermal-resistant cellulose-based composite nonwoven as lithium-ion battery separator. ACS Appl. Mater. Interfaces.

[25] Yanilmaz M., Lu Y., Zhu J., Zhang X. (2016). Silica/polyacrylonitrile hybrid nanofiber membrane separators via sol-gel and electrospinning techniques for lithium-ion batteries. J. Power Sources.

[26] Kim H.Y., Cho Y., Kang S.W. (2019). Porous Cellulose acetate membranes prepared by water pressure-assisted process for water-treatment. J. Ind. Eng. Chem..

[27] Lee W.G., Kang S.W. (2019). Control of pore in cellulose acetate containing Mg salt by water pressure treatment for applications to separators. J. Ind. Eng. Chem..

[28] Lee W.G., Kang S.W. (2020). Eco-friendly process for facile pore control in thermally stable cellulose acetate utilizing zinc(II) nitrate for water-treatment. J. Ind. Eng. Chem..

[29] Lee H.J., Cho Y., Kang S.W. (2021). Development of low-cost process for pore generation in cellulose acetate by utilizing calcium salts. J. Ind. Eng. Chem..

[30] Jie S., Tong S., He Z., Yang R. (2017). Recent developments of cellulose materials for lithium-ion battery separators. Cellulose.

[31] Lizundia E., Costa C.M., Alves R., Méndez S.L. (2020). Cellulose and its derivatives for lithium ion battery separators: A review on the processing methods and properties. Carbohydr. Polym. Technol. Appl..

[32] Pan R., Cheung O., Wang Z., Tammela P., Huo J., Lindh J., Edström K., Strømme M., Nyholm L. (2016). Mesoporous Cladophora cellulose separators for lithium-ion batteries. J. Power Sources.

[33] Hong S.H., Cho Y., Kang S.W. (2021). Formation of water-channel by propylene glycol into polymer for porous materials. Membranes.

[34] Lee W.G., Kim D.H., Jeon W.C., Kwak S.K., Kang S.J., Kang S.W. (2017). Facile control of nanoporosity in cellulose acetate using Nickel (II) nitrate additive and water pressure treatment for highly efficient battery gel separators. Sci. Rep..

